# Supplementary material for: Content-rich biological network constructed by mining PubMed abstracts
Source: BMC Bioinformatics. 2004 Oct 8;5:147. doi: 10.1186/1471-2105-5-147 (PMC528731; doi:10.1186/1471-2105-5-147)
Supplement: Additional File 2 — The original results of the above study (non-essential files are deleted to keep the file size under the limit set by BMC bioinformatics). [file 1471-2105-5-147-S2.bz2 › chilibotAdditionalFile2/dip30/377ID9927421E9790/html/NGFR_NTRK1.html]

 


 **NGFR** and **NTRK1** 
  
Found 541 abstracts in PubMed, retrieved 30.  
 

 What does Google say? 
 PDF only 
| .edu only 

---

**Interactive relationship** (e.g. stimulation, inhibition, etc)

**Stimulatory relationship**- To address this issue, various combinations of transfected PC12 cells expressing a PDGFR TrkA  [ **NTRK1** ]  chimera and the p75NTR  [ **NGFR** ]  selective NGF mutant D9 13 NGF were utilized to selectively stimulate TrkA  [ **NTRK1** ]  or p75NTR  [ **NGFR** ]  signaling, respectively.  Ref: 12702729 J Biol Chem< MedlineTA>J Biol Chem,
- On the one hand, p75NTR  [ **NGFR** ]  provides a positive modulatory influence on nerve growth factor NGF signaling through the high affinity neurotrophin receptor TrkA  [ **NTRK1** ] , and hence increases NGF survival signaling.  Ref: 12453482 Neuroscience, 2002
- While activation of TrkA  [ **NTRK1** ]  signaling is sufficient for eliciting neurite outgrowth in PC12 cells, signaling through p75NTR  [ **NGFR** ]  plays a modulatory role, especially in the increased formation of fine, synaptic bouton like structures, in which both TrkA  [ **NTRK1** ]  and p75NTR  [ **NGFR** ]  appear to co localize.  Ref: 12702729 J Biol Chem< MedlineTA>J Biol Chem, 2002
**Neutral relationship**- Adrenergic mouse pheochromocytoma MPC cells from heterozygous neurofibromatosis knockout mice show little or no expression of the NGF receptor  [ **NGFR** ]  trk A  [ **NTRK1** ]  and do not undergo neuronal differentiation in response to NGF.  Ref: 12438155 Ann N Y Acad Sci, 2002
- Overall, our results indicate an essential role for p75NTR  [ **NGFR** ]  in supporting NGF triggered TrkA  [ **NTRK1** ]  signaling pathways mediating neuronal survival in hippocampal neurons.  Ref: 12453482 Neuroscience, 2002
- In addition, a new interaction in the TrkA  [ **NTRK1** ]  p75NTR  [ **NGFR** ]  heteromeric receptor signal transduction network was revealed, namely that NGF induced activation of the MAPK pathway appears to inhibit the parallel NGF induced Akt pathway.  Ref: 12702729 J Biol Chem< MedlineTA>J Biol Chem, 2002
- Although neurotrophins did not regulate p75NTR  [ **NGFR** ]  processing, the alpha and gamma secretase mediated cleavage of p75 is modulated by receptor tyrosine kinases Trks TrkA  [ **NTRK1** ]  and TrkB but not TrkC.  Ref: 12843241 J Neurosci, 2003
- A chimera of the nerve growth factor NGF receptor  [ **NGFR** ] , TrkA  [ **NTRK1** ] , and green fluorescent protein GFP was engineered by expressing GFP in phase with the carboxyl terminus of TrkA  [ **NTRK1** ] .  Ref: 12438306 J Biol Chem, 2003
- Previous studies from this laboratory and others have suggested the evidences that acidic glycosphingolipid, ganglioside GM1 GM1, is an endogenous regulator of high affinity nerve growth factor receptor  [ **NTRK1** ]   [ **NGFR** ] , Trk  [ **NTRK1** ] , which is an essential factor for the normal development and differentiation of neuronal cells by forming a complex with Trk  [ **NTRK1** ] .  Ref: 12374216 Neurochem Res, 2002

**Non-interactive relationship** (e.g. studied together, co-existance, homology, etc.)

- PURPOSE The purpose is to analyze the possible correlation between expression and activation of the high affinity nerve growth factor NGF receptor  [ **NGFR** ]  TrkA  [ **NTRK1** ] , cell cycle protein expression, and disease outcome in serous ovarian carcinoma.  Ref: 12796393 Clin Cancer Res< MedlineTA>Clin Cancer Res, 2002
- In the present study, the effect of signaling generated from the Erb B2 homodimer as a result of transcription of the MUC1 gene was investigated in human mammary epithelial cell lines MTSV1 7 and Hb2 stably transfected with a pBAT trk  [ **NTRK1** ]  neu construct in which the extracellular domain of Erb B2 was replaced with the corresponding domain from the nerve growth factor NGF receptor  [ **NGFR** ] .  Ref: 12798775 Cell Biol Int, 2003
- Here we show that survival of sympathetic neurons supported by NGF on distal axons requires the kinase activity of the NGF receptor  [ **NGFR** ] , TrkA  [ **NTRK1** ] , in both distal axons and cell bodies.  Ref: 12848932 Neuron, 2003
- SHB mediates certain responses in platelet derived growth factor PDGF receptor, fibroblast growth factor FGF receptor, neural growth factor NGF receptor  [ **NGFR** ]  TRKA  [ **NTRK1** ] , T cell receptor, interleukin 2 IL 2 receptor and focal adhesion kinase FAK signaling.  Ref: 12776987 Curr Mol Med, 2003
- Expression and Activation of the Nerve Growth Factor Receptor  [ **NGFR** ]  TrkA  [ **NTRK1** ]  in Serous Ovarian Carcinoma.  Ref: 12796393 Clin Cancer Res< MedlineTA>Clin Cancer Res, 2003
- P75NTR  [ **NGFR** ]  signaling independent of TrkA  [ **NTRK1** ] , such as increased neutral sphingomyelinase NSMase activity causing enhanced levels of ceramide, were not detected after exposure of hippocampal neurons to NGF.  Ref: 12453482 Neuroscience, 2002
- A long standing question in neurotrophin signal transduction is whether heteromeric TrkA  [ **NTRK1** ]  p75NTR  [ **NGFR** ]  complexes possess signaling capabilities that are significantly different from homo oligomeric TrkA  [ **NTRK1** ]  or p75NTR  [ **NGFR** ]  alone.  Ref: 12702729 J Biol Chem< MedlineTA>J Biol Chem, 2002
- However, p75NTR  [ **NGFR** ]  may also signal independently of TrkA  [ **NTRK1** ] , causing cell death or cell survival, depending on the cell type and stage of development.  Ref: 12453482 Neuroscience, 2002
- However, NGF rescue from growth factor withdrawal requires NGF signalling through the high affinity tyrosine kinase receptor  [ **NTRK1** ]  TrkA  [ **NTRK1** ] , while NGF rescue from TNFalpha promoted cell death requires NGF signalling through the low affinity p75NTR  [ **NGFR** ]  receptor.  Ref: 12882321 Aging Cell, 2003
- 5 immunohistochemistry study revealed that the expression of nerve growth factor receptor  [ **NGFR** ]  trk A  [ **NTRK1** ]  was induced by acupuncture after cerebral ischemia, which may play a role in antagonizing apoptosis.  Ref: 12532826 Sheng Li Ke Xue Jin Zhan, 1999
- Individual and combined effects of TrkA  [ **NTRK1** ]  and p75NTR  [ **NGFR** ]  nerve growth factor NGF receptors A role for the high affinity receptor site.  Ref: 12702729 J Biol Chem< MedlineTA>J Biol Chem, 1999
- Quantitative in situ hybridization was used to detect changes in neuronal PACAP mRNA expression and to correlate this expression with neuronal mRNA expression of the NGF receptor  [ **NGFR** ]  tyrosine kinase trk A  [ **NTRK1** ] .  Ref: 12890505 Neuroscience, 2003
- The contrary response of different cells to neurotrophins in terms of cell cycle regulation derives in part from the fact that these factors use two different receptor types to transmit their signals members of the Trk  [ **NTRK1** ]  family and the p75 neurotrophin receptor p75NTR  [ **NGFR** ] .  Ref: 12371150 Histol Histopathol, 2002
- Astrocytes have been implicated in axon guidance and synaptic regeneration in the retina and these processes involve activation of the high affinity nerve growth factor receptor  [ **NTRK1** ]   [ **NGFR** ] , known as the tyrosine kinase A receptor.  Ref: 12536040 Neurosci Lett, 2003
- OBJECTIVE We aimed to investigate the expression of nerve growth factor NGF and high affinity NGF receptor  [ **NGFR** ]  p140 TrkA  [ **NTRK1** ]  on chondrocytes from human healthy and osteoarthritic cartilage.  Ref: 12468822 Rheumatology Oxford , 2002
- Pathways responsible for transducing the sensitisation of TRPV1 by TrkA  [ **NTRK1** ] , the NGF receptor  [ **NGFR** ] , were characterised by observing the effects of inhibitors of key members of NGF activated second messenger signalling cascades.  Ref: 12815188 J Physiol< MedlineTA>J Physiol, 2002
- Mouse PTPRO mRNA was observed in neuron populations expressing TrkA  [ **NTRK1** ] , the high affinity nerve growth factor receptor  [ **NTRK1** ]   [ **NGFR** ] , or TrkC, the neurotrophin 3 receptor, and immunoreactive mPTPRO and TrkC colocalized in large dorsal root ganglia proprioceptive neurons.  Ref: 12532410 J Comp Neurol, 2003
- Increased expression of nerve growth factor NGF and high affinity NGF receptor  [ **NGFR** ]  p140 TrkA  [ **NTRK1** ]  in human osteoarthritic chondrocytes.  Ref: 12468822 Rheumatology Oxford , 2002
- RESULTS These results revealed a low amount of NGF in the plasma of old subjects associated with a reduced expression of mRNA for NGF receptor  [ **NGFR** ]  trkA  [ **NTRK1** ]  in peripheral lymphocytes.  Ref: 12679610 Gerontology< MedlineTA>Gerontology, 2002
- There was no detectable expression of high affinity tyrosine kinase receptor  [ **NTRK1** ]  trkA  [ **NTRK1** ]   **NGFR** .  Ref: 12580757 J Mol Endocrinol, 2003
- In the older GK rats, both mRNA and protein expression of nerve growth factor in the sciatic nerve were significantly reduced, and  **NGFR**  TrkA  [ **NTRK1** ]  and NGFRp75 protein expression was reduced in dorsal root ganglia.  Ref: 12469361 Diabetes Metab Res Rev< MedlineTA>Diabetes Metab Res Rev, 2003
- In mouse hippocampal cells lacking p75NTR  [ **NGFR** ]  p75NTR  [ **NGFR** ]  activation of TrkA  [ **NTRK1** ]  by NGF was not detectable.  Ref: 12453482 Neuroscience, 2002
- METHODS Retroviral vectors were constructed to express the high affinity NGF receptor  [ **NGFR** ]  TrkA  [ **NTRK1** ]  and low affinity NGF receptor  [ **NGFR** ]  p75 NTR.  Ref: 12877805 Chin Med J (Engl), 2003
- Thus, a chimeric receptor was created that possessed the extracellular and transmembrane domains of the nerve growth factor NGF receptor  [ **NGFR** ]  TrkA  [ **NTRK1** ]  and the cytoplasmic domain of PDGFRbeta TTbeta.  Ref: 12794304 J Mol Neurosci, 2003
- After 2 weeks of treatment of PLA cells with isobutylmethylxanthine, indomethacin, and insulin, about 20 to 25 percent of the cells differentiated into cells with typical neural morphologic characteristics, accompanied by increased expression of NSE, vimentin, and the nerve growth factor receptor  [ **NGFR** ]  trk A  [ **NTRK1** ] .  Ref: 12711954 Plast Reconstr Surg, 2003
- CONCLUSIONS This is the first study showing that human chondrocytes synthesize NFG beta and express on their surface the high affinity  **NGFR**  p140 TrkA  [ **NTRK1** ] .  Ref: 12468822 Rheumatology Oxford , 2002
- The neurotrophins are able to bind to two different receptors all bind to a common receptor p75NTR  [ **NGFR** ] , and each also binds to one of a family of Trk  [ **NTRK1** ]  receptors.  Ref: 12787319 Neuropathol Appl Neurobiol, 2003
